# *Toxoplasma gondii* Infections and Associated Factors in Female Children and Adolescents, Germany

**DOI:** 10.3201/eid3005.231045

**Published:** 2024-05

**Authors:** Laura Giese, Frank Seeber, Anton Aebischer, Ronny Kuhnert, Martin Schlaud, Klaus Stark, Hendrik Wilking

**Affiliations:** Robert Koch Institute, Berlin, Germany

**Keywords:** *Toxoplasma gondii*, toxoplasmosis, seroprevalence, seroepidemiologic studies, parasitic diseases, congenital toxoplasmosis, cross-sectional studies, children, adolescents, risk factors, Germany

## Abstract

In a representative sample of female children and adolescents in Germany, *Toxoplasma gondii* seroprevalence was 6.3% (95% CI 4.7%–8.0%). With each year of life, the chance of being seropositive increased by 1.2, indicating a strong force of infection. Social status and municipality size were found to be associated with seropositivity.

Toxoplasmosis, caused by the protozoan parasite *Toxoplasma gondii,* is the most common parasitic foodborne disease in Germany. The seroprevalence in adults is exceptionally high (50%) compared with other countries (*1*). After infection via either undercooked meat from infected animals (pathway 1) or uptake of infectious oocysts shed by infected cats (pathway 2), *T. gondii* persists lifelong in infected persons, posing a risk for reactivation of latent infections (*2*). The proportion of pathways 1 and 2 leading to infection is largely unknown. We previously argued that in Germany, eating habits like consumption of raw pork products are likely responsible for the high seroprevalence (*1*). Infections in immune-competent persons remain largely asymptomatic or cause only mild, influenza-like symptoms. However, severe disease manifestations can occur, including ocular toxoplasmosis with sequalae, severe and often fatal consequences in immunocompromised persons, and congenital toxoplasmosis (*2*).

Worldwide, the World Health Organization (WHO) considered the burden of disease from toxoplasmosis to be high. Nevertheless, routine disease and pathogen surveillance is inadequate, so the incidence of human infection and parasite occurrence in animals and food is underestimated (*3*). In Germany, *T. gondii* screening during pregnancy to detect and treat primary infection, which could prevent parasite transmission to the unborn, is not covered by the statutory health insurance. Ongoing discussions to reevaluate this policy demand data specifically assessing risk estimates for women of reproductive age.

In adults, age, dietary habits, and region of residence are associated with seroprevalence (*1*). Comparable analyses for children and adolescents are missing but are needed to estimate the public health problem and to suggest countermeasures. To provide such baseline data, this study aimed to estimate seroprevalence and to determine associated factors of *T. gondii* infections in female children and adolescents in Germany. The Hannover Medical School ethics committee approved the study (*4*).

## The Study

The second wave of the nationwide German Health Interview and Examination Survey for Children and Adolescents (KiGGS Wave 2) was conducted as a 2-stage sampling survey during 2014‒2017 in 167 representative sample points in Germany (*4*). Serum specimens of participants 3 ‒17 years of age were tested for the presence of *T. gondii* IgG using a highly sensitive and specific commercial enzyme linked fluorescence assay (ELFA), as described previously (*1*,*5*).

We determined overall and stratified seroprevalence. We used data from standardized interviews to assess factors associated with seropositivity. On the basis of our previous serosurvey results in adults (*1*), we tested potential associations between seropositivity and vegetarianism, residence (eastern or western Germany), municipality size, and socioeconomic status. We identified minimal adjustment sets for multivariable logistic regression models by using directed acyclic graphs (Appendix). We determined adjusted odds ratios (aOR) for each exposure variable with 95% CIs. We used sampling weights for all statistical analyses accounting for the study design. In addition, we calculated survey weights based on age, sex, residence, nationality, and education to correct for deviations from national population statistics. 

We included 1,453 girls and adolescents (mean age 10.3 years) in the analyses. Of those, 1,359 tested negative and 94 tested positive for *T. gondii*‒specific IgG. We estimated overall weighted seroprevalence at 6.3% (95% CI 4.7%–8.0%) (Table).

**Table:** Stratified weighted seroprevalence of *Toxoplasma gondii* and results of weighted logistic regression analyses of factors potentially associated with seropositivity in female children and adolescents, Germany, 2014–2017*

Characteristics	No. positive/no. total†	Prevalence (95% CI)	Multivariable analysis aOR (95% CI)
Age group, y			
3‒6	11/272	3.5 (1.3–5.8)	Referent
7‒10	11/339	3.0 (0.8–5.1)	0.8 (0.3–2.3)
11‒13	23/363	7.0 (3.5–10.4)	2.0 (0.9–4.9)
14 ‒17	49/481	11.3 (6.7–15.8)	3.5 (1.6–7.4)
Socioeconomic status‡			
Low	20/211	10.8 (4.7–16.9)	2.7 (1.3–5.9)
Middle	51/868	4.7 (3.1–6.3)	Referent
High	19/325	6.1 (3.1–9.2)	1.5 (0.7–3.0)
Municipality size§			
<5,000	26/308	9.1 (4.4–13.7)	2.6 (1.1–5.7)
5,000 to <20,000	24/411	4.0 (1.9–6.1)	Referent
20,000 to <100,000	23/401	6.4 (2.8–9.9)	1.9 (0.8–4.5)
>100,000	21/333	6.7 (3.9–9.5)	2.2 (1.1–4.4)
Residence¶			
East	57/954	6.3 (4.3–8.3)	Referent
West	37/499	6.5 (4.0–8.9)	1.1 (0.6–1.8)
Vegetarian			
Yes	7/129	6.4 (4.6–8.2)	1.3 (0.5–3.3)
No	86/1,273	7.4 (1.3–13.5)	Referent
Total	94/1,453	6.3 (4.7–8.0)	

*We tested 5 hypothesized associations toward seropositivity. Minimum adjustment sets were identified based on directed acyclic graphs (Appendix). aOR, adjusted odds ratio. †Unweighted. ‡Based on the multidimensional socioeconomic index according to Lampert et al. (*4*). §By number of inhabitants. ¶Western states: Baden-Württemberg, Bavaria, Bremen, Hamburg, Hesse, Lower Saxony, North Rhine-Westfalia, Rhineland-Palatinate, Saarland, Schleswig-Holstein; Eastern states: Berlin, Brandenburg, Mecklenburg-West Pomerania, Saxony, Saxony-Anhalt, Thuringia.

**Figure 1:**
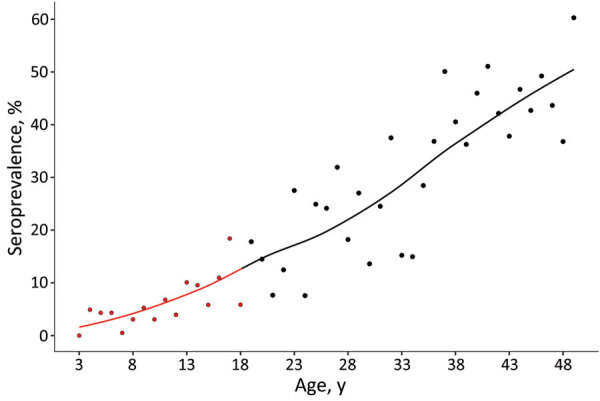
Weighted seroprevalence of *Toxoplasma gondii* infections in female children and adolescents by age, Germany, 2014–2017 (red). For comparison, results of Wilking et al. (*3*), a previous study among adults, were added to the graph (black)

With each year of life, the chance of being seropositive increased significantly, by 1.2 (95% CI 1.1–1.3). When we combined the data from the girls with those of female adults, seroprevalence steadily increased with age (Figure 1). This increase is a result of cumulative seropositivity because *T. gondii* infection is persistent and shows little seroreversion.

Girls living in families with low socioeconomic status (SES) showed the highest prevalence (10.8%, 95% CI 4.7%–16.9%). Their chance of being seropositive is 2.7 (95% CI 1.3–5.9) times higher than that of girls with middle SES. Low social status is often found to be associated with various disease risks, which may be a result of lower health literacy and reduced options to avoid health-related risks (*4*).

**Figure 2:**
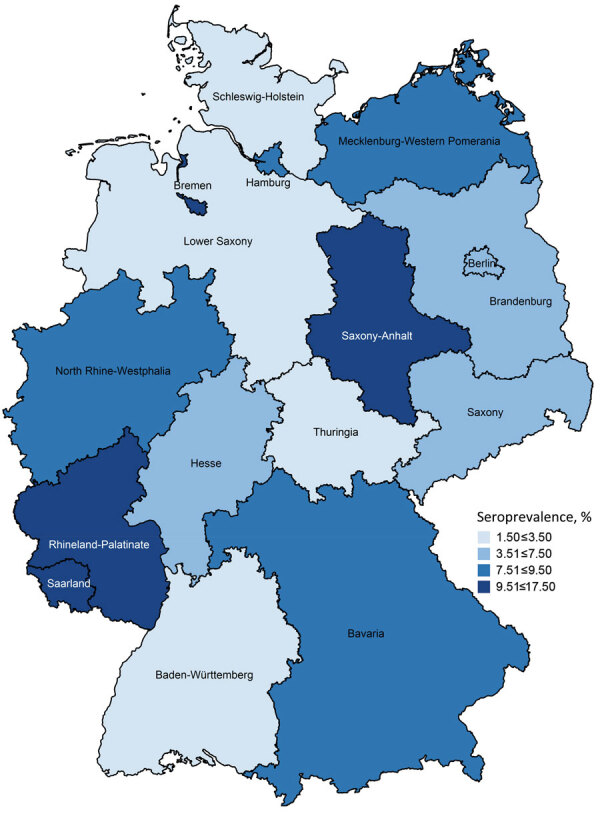
Weighted seroprevalence of *Toxoplasma gondii* infections in female children and adolescents by federal state, Germany, 2014–2017.

The seroprevalence among girls living in rural areas (<5,000 inhabitants) was significantly higher than seroprevalence for girls living in small towns (5,000–<20,000 inhabitants) (aOR 2.6, 95% CI 1.1–5.7). Similarly, girls living in urban areas (>100,000 inhabitants) were also 2.2 (95% CI 1.1–4.4) times more likely to be seropositive than those living in small towns. Greater exposure to natural habitats, including sand and soil contaminated due to free-roaming cats (*6*), as well as cats and children using the same limited spaces (e.g., sandboxes) (*7*), might explain the respective higher risks of infection. We did not observe any regional distribution patterns (e.g., differences between eastern and western Germany) (Figure 2).

Overall, 9.3% of the participants reported being vegetarian; of those, 6.4% (95% CI 4.6%–8.2%) were seropositive (aOR 1.3, 95% CI 0.5–3.3). This result is not significantly different from results for nonvegetarians and is consistent with other studies (*8*–*10*). Diet appeared to have less effect on the risk for infection in children and adolescents than in adults. This finding may indicate that the relevance of the 2 transmission pathways differs significantly between age groups, and more environmentally associated infections occur in children and adolescents. Alternatively, risk factors associated with transmission pathway 1 may have shifted over time, for example, from improvements in the production and preparation of meat. However, more detailed information on the type (raw or undercooked) and quantity of meat consumed would have been beneficial but were not available in our dataset.

## Conclusions

Overall, 6 of every 100 girls in Germany become infected with *T. gondii* during the first 18 years of life, corresponding to ≈340,504 total infections in this population group. Internationally comparable studies are limited. Seroprevalence estimates vary worldwide, from <10% to >60% for girls and 10%–80% for adults (*11*,*12*). Toxoplasmosis causes a higher infection pressure for girls and young women in Germany than in countries with similar socioeconomic conditions (*11*,*13*). In the United States, for example, the seroprevalence is significantly lower in age groups 6‒11 years (0.9%, 95% CI 0.5%–1.5%) and 12‒19 years (3.1%, 95% CI 2.0%–4.6%) (*11*).

Independent risk factors identified in our study were age, low social status, and growing up in rural or urban areas. Those factors have also been associated with seropositivity in children and adolescents in other countries (*12*,*14*). Further risk factors include contact with cats (*12*,*14*,*15*), contact with soil or sand (*8*,*9*,*15*), and consumption of unwashed vegetables (*8*). Growing up on a farm or keeping farm animals was also associated with increased seroconversions (*8*,*10*). Regular handwashing showed protective effects (*9*,*15*).

Our data may be helpful as an empirical basis for prevention guidelines. Implementing screening of pregnant women is one possibility and should be reevaluated using current data. Furthermore, our results indicate that meat consumption does not appear to be a driving force in children and adolescents, which calls for different prevention strategies in this population than in adults (Appendix). However, our serosurvey is cross-sectional and represents the cumulated lifetime risk for infection. Therefore, misclassification of exposures (e.g., participants reported vegetarianism but consumed raw meat earlier in life) and unmeasured confounding is likely. Thus, our data are not appropriate for establishing causal relationships. Future studies should use longitudinal data containing detailed information on exposures and time of infection to disentangle different transmission pathways. The ultimate goal is efficient primary prevention of *T. gondii* infections; the goal requires integrating the fields of veterinary, human, and environmental medicine in a One Health approach.

Appendix Additional information about *Toxoplasma gondii* infections and associated factors in female children and adolescents, Germany.
