# Supplementary material for: Toxoplasma gondii Infections and Associated Factors in Female Children and Adolescents, Germany
Source: Emerg Infect Dis. 2024 May;30(5):995–9. doi: 10.3201/eid3005.231045 (PMC11060440; doi:10.3201/eid3005.231045)
Supplement: Appendix — Additional information about Toxoplasma gondii infections and associated factors in female children and adolescents, Germany. [file 23-1045-Techapp-s1.pdf]

Article DOI: http://doi.org/10.3201/eid3005.231045

*EID cannot ensure accessibility for supplementary materials supplied by authors. Readers who have difficulty accessing supplementary content should contact the authors for assistance.*

# *Toxoplasma gondii* Infections and Associated Factors in Female Children and Adolescents, Germany

## Appendix

### Additional Discussion: Practical recommendations for the prevention of *Toxoplasma gondii*

To effectively prevent toxoplasmosis, the various transmission routes and risk factors must be considered in a One Health approach combining the fields of human, animal and environmental health. There are currently three main opportunities for prevention:

Opportunity 1: Regarding congenital toxoplasmosis, regular testing during pregnancy is the only way to timely detect and treat infections in the unborn child. The implementation of mandatory screening programs during pregnancy should therefore be systematically evaluated. This includes the specificity and sensitivity of diagnostic tests and the consequences of a positive test result, including treatment options and effects (*1*).

Opportunity 2: Population groups that may be at a higher risk of primary infection and disease (e.g., pregnant women, people with low social status or people living in rural/urban areas) should be particularly addressed in health education programs. Although not apparent from our results, cat owners and raw meat consumers should also be targeted (*2*). The overall aim should be to increase the level of knowledge about toxoplasmosis, its transmission and prevention. Also, the use of new media should be considered to specifically address children and adolescents.

Practical recommendations should include how to reduce the risk of infection from the environment, for example by i. following hygiene measures when cleaning cat litter or when being in contact with sand or soil (e.g., handwashing or wearing gloves), ii. feeding cats with commercial food, iii. keeping cats away from public playgrounds or sand boxes. They should also include how to prevent foodborne infections, for example by i. avoiding the consumption of raw or undercooked meat or by ii. washing fruits and vegetables before consumption (*3*).

Opportunity 3: Measures to reduce the spread of *T. gondii* in livestock and the environment, for example through rodent control, monitoring of livestock and freshly produced meat or parasite inactivation, are discussed by experts of biologic hazards (*4*). These have to be evaluated and implemented in interdisciplinary cooperation between infectious disease experts and experts in environmental and veterinary health.

### Additional References


1. Ahmed M, Sood A, Gupta J. Toxoplasmosis in pregnancy. Eur J Obstet Gynecol Reprod Biol. 2020;255:44–50. PubMed https://doi.org/10.1016/j.ejogrb.2020.10.003

2. Wilking H, Thamm M, Stark K, Aebischer T, Seeber F. Prevalence, incidence estimations and risk factors of *Toxoplasma gondii* infection in Germany: a representative, cross-sectional, serological study. Sci Rep. 2016;6:22551. PubMed https://doi.org/10.1038/srep22551

3. Centers for Disease Control and Prevention. Parasites – Toxoplasmosis (Toxoplasma infection). 2018 Sep 27 [cited 2024 Feb 14]. https://www.cdc.gov/parasites/toxoplasmosis/

4. EFSA Panel on Biological Hazards (BIOHAZ). Koutsoumanis K, Allende A, Alvarez-Ordónez A, Bolton D, Bover-Cid S, Chemaly M, et al. Public health risks associated with food-borne parasites. EFSA J. 2018;16:5495.


## Directed Acyclic Graphs

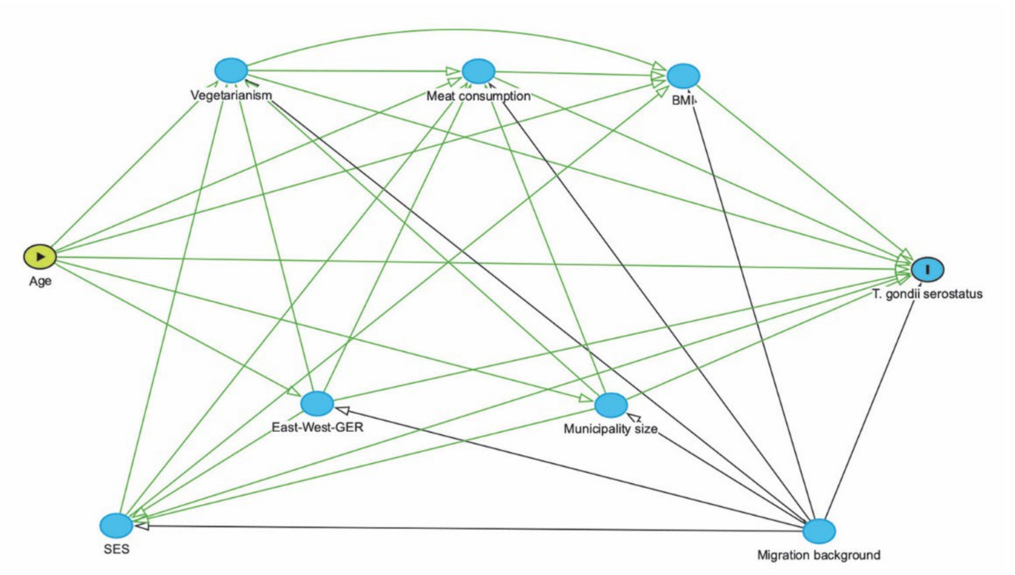


**Appendix Figure 1.** Directed Acyclic Graph (DAG) used to select the minimum adjustment set in the analysis of the hypothesized association between age and seropositivity in female children and adolescents in Germany. The graph shows correlations between variables included in our dataset based on current evidence. Green paths represent causal paths, red paths are biasing paths for which we have adjusted in our multivariable model. The DAG was created using Dagitty.net.

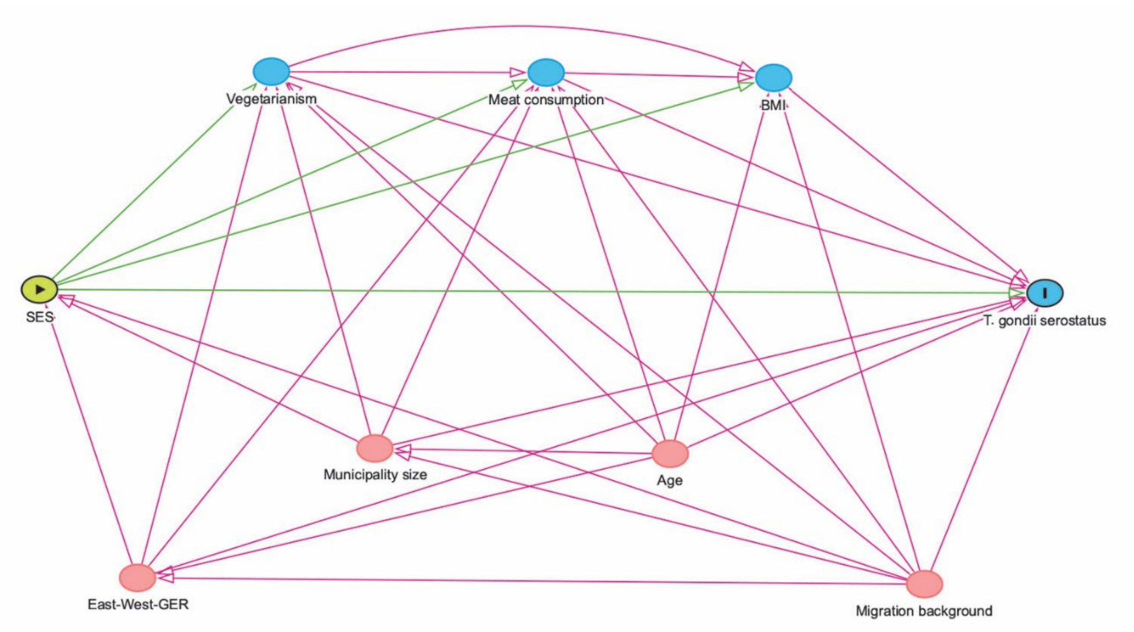


**Appendix Figure 2.** Directed Acyclic Graph (DAG) used to select the minimum adjustment set in the analysis of the hypothesized association between socioeconomic status (SES) and seropositivity in female children and adolescents in Germany. The graph shows correlations between variables included in our dataset based on current evidence. Green paths represent causal paths, red paths are biasing paths for which we have adjusted in our multivariable model. The DAG was created using Dagitty.net.

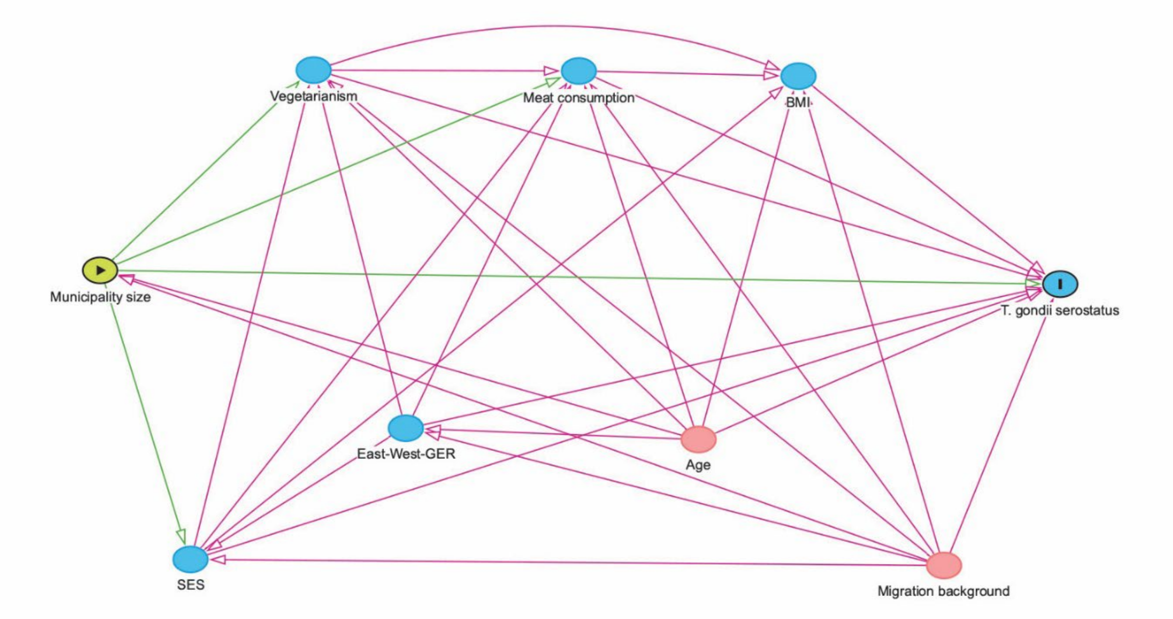


**Appendix Figure 3.** Directed Acyclic Graph (DAG) used to select the minimum adjustment set in the analysis of the hypothesized association between municipality size and seropositivity in female children and adolescents in Germany. The graph shows correlations between variables included in our dataset based on current evidence. Green paths represent causal paths, red paths are biasing paths for which we have adjusted in our multivariable model. The DAG was created using Dagitty.net.

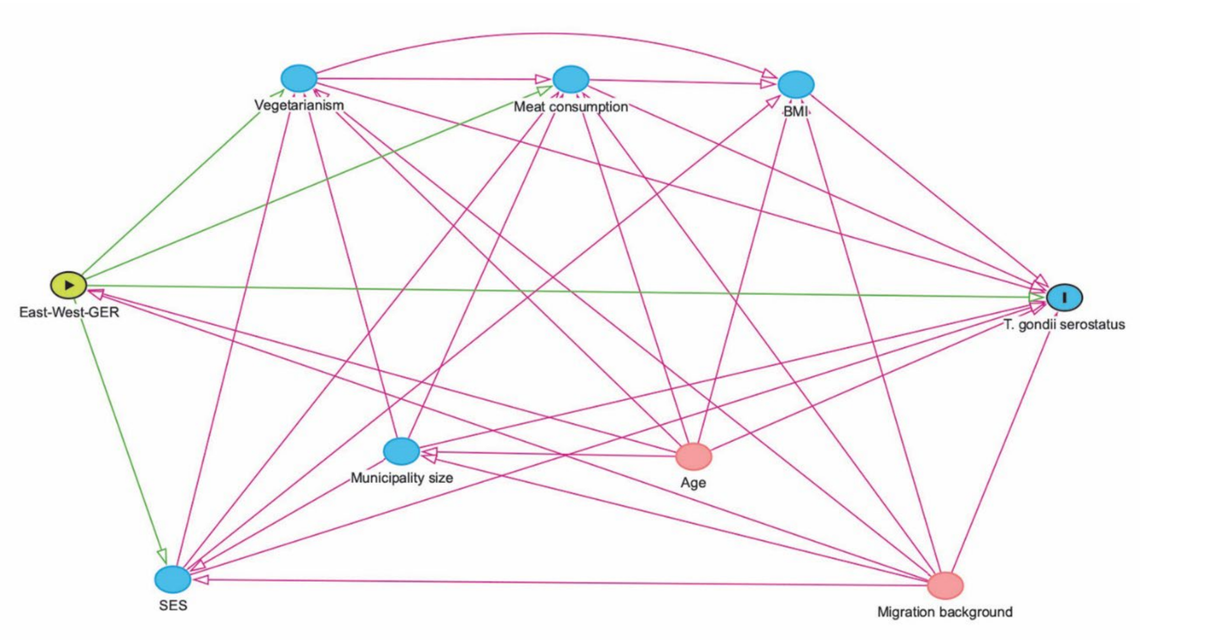


**Appendix Figure 4.** Directed Acyclic Graph (DAG) used to select the minimum adjustment set in the analysis of the hypothesized association between East-West-Germany (East-West-GER) and seropositivity in female children and adolescents in Germany. The graph shows correlations between variables included in our dataset based on current evidence. Green paths represent causal paths, red paths are biasing paths for which we have adjusted in our multivariable model. The DAG was created using Dagitty.net.

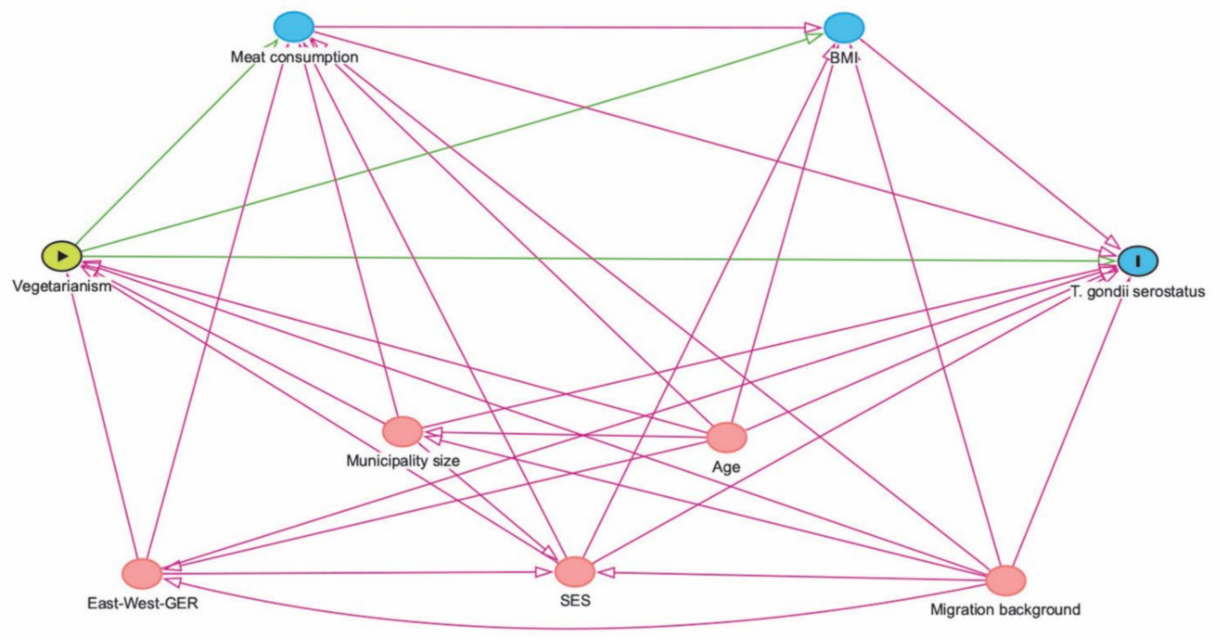


**Appendix Figure 5.** Directed Acyclic Graph (DAG) used to select the minimum adjustment set in the analysis of the hypothesized association between Vegetarianism and seropositivity in female children and adolescents in Germany. The graph shows correlations between variables included in our dataset based on current evidence. Green paths represent causal paths, red paths are biasing paths for which we have adjusted in our multivariable model. The DAG was created using Dagitty.net.
